# Molecular Characteristics and Incidence of Apple Rubbery Wood Virus 2 and Citrus Virus A Infecting Pear Trees in China

**DOI:** 10.3390/v14030576

**Published:** 2022-03-11

**Authors:** Yanxiang Wang, Ying Wang, Guoping Wang, Qingyu Li, Zhe Zhang, Liu Li, Yuzhuo Lv, Zuokun Yang, Jiashu Guo, Ni Hong

**Affiliations:** 1 Key Lab of Plant Pathology of Hubei Province, College of Plant Science and Technology, Huazhong Agricultural University, Wuhan 430070, China; wangyanxiang@webmail.hzau.edu.cn (Y.W.); heiha1820@163.com (Y.W.); gpwang@mail.hzau.edu.cn (G.W.); zhangzz0618@163.com (Z.Z.); 15032210591@163.com (L.L.); yuzhuolv@163.com (Y.L.); 13297974203@163.com (Z.Y.); guojiashu94@163.com (J.G.); 2 Hubei Key Laboratory of Edible Wild Plants Conservation and Utilization, College of Life Sciences, Hubei Normal University, Huangshi 435002, China; 3 Yantai Academy of Agricultural Science, Yantai 264000, China; liqingyu613891@163.com; 4 Key Laboratory of Horticultural Crop (Fruit Trees) Biology and Germplasm Creation of the Ministry of Agriculture, Huazhong Agricultural University, Wuhan 430070, China

**Keywords:** apple rubbery wood virus 2, citrus virus A, genome, subcellular localization, pear

## Abstract

Apple rubbery wood virus 2 (ARWV-2) and citrus virus A (CiVA) belong to a recently approved family *Phenuiviridae* in the order *Bunyavirales* and possess negative-sense single-stranded RNA genomes. In this study, the genome sequence of three ARWV-2 isolates (S17E2, LYC2, and LYXS) and a CiVA isolate (CiVA-P) infecting pear trees grown in China were characterized using high-throughput sequencing combined with conventional reverse-transcription PCR (RT-PCR) assays. The genome-wide nt sequence identities were above 93.6% among the ARWV-2 isolates and above 93% among CiVA isolates. Sequence comparisons showed that sequence diversity occurred in the 5′ untranslated region of the ARWV-2 genome and the intergenic region of the CiVA genome. For the first time, this study revealed that ARWV-2 proteins Ma and Mb displayed a plasmodesma subcellular localization, and the MP of CiVA locates in cell periphery and can interact with the viral NP in bimolecular fluorescence complementation assays. RT-PCR tests disclosed that ARWV-2 widely occurs, while CiVA has a low incidence in pear trees grown in China. This study presents the first complete genome sequences and incidences of ARWV-2 and CiVA from pear trees and the obtained results extend our knowledge of the viral pathogens of pear grown in China.

## 1. Introduction

Apple rubbery wood virus 1 (ARWV-1) together with apple rubbery wood virus 2 (ARWV-2) were discovered in apple trees showing rubbery wood disease symptoms in Germany and USA [1]. Both ARWV-1 and ARWV-2 possess a tripartite negative-sense single-stranded RNA (-ssRNA) genome and are associated with apple rubbery wood disease [1]. The complementary RNA (cRNA) of each genomic RNA contains one open reading frame (ORF), with RNAs1–3 (large (L), medium (M), and small (S)) encoding an RNA-dependent RNA polymerase (RdRp), a movement protein (MP), and a nucleocapsid protein (NP), respectively. The major difference between ARWV-1 and ARWV-2 is that their encoded proteins have identities ranging from 59% (RdRp proteins) to ~66–68% (NP proteins). In addition, a few isolates of ARWV-2 have two distinct M segments and two distinct S RNA segments, referred as ARWV 2 Ma, 2 Mb, 2 Sa, and 2 Sb. The 2 Ma and 2 Mb segments have nt sequence identity of ~66%, and 2 Sa and 2 Sb segments have nt sequence identity of ~56% [1]. According to the genomic structures, phylogenetic relationships and coding protein features with those of viruses in the *Bunyavirales* order, the two viruses with species names *Apple rubodvirus 1* and *Apple rubodvirus 2* are assigned to the genus *Rubodvirus* in the recently approved family *Phenuiviridae* in the *Bunyavirales* order. In addition, the genus *Rubodvirus* contains two other virus species *Grapevine rubodvirus 1* and *Grapevine rubodvirus 2* identified from grapevine [2]. Recently, ARWV-1 and ARWV-2 have been identified in apple grown in China [3,4], and ARWV-2 was reported to be able to infect pear in China [5] and apple in Italy [6]. ARWV-1 and ARWV-2 together with citrus concave gum-associated virus (CCGaV) were also identified in apple-decline-afflicted samples in the United States and apple trees from a global apple collection [7].

Citrus virus A (CiVA) was described in a non-symptomatic, field-grown sweet orange tree in southern Italy [8]. The CiVA genome consists of two -ssRNA segments, with a large RNA (RNA1) encoding a RdRp in its complementary RNA and an ambisense RNA (RNA2) encoding MP and NP. The species name *Coguvirus eburi* has been established for CiVA, and the virus belongs to genus *Coguvirus* in the recently approved family *Phenuiviridae* [9]. The genus *Coguvirus* contains three species, including the type species *Citrus coguvirus* (the species name of CCGaV), *Coguvirus eburi,* and *Grapevine coguvirus* (the species name of grapevine associated cogu-like virus 1, GaCLV-1) [2,10]. Now, CiVA has been identified in pear in France [11], and is also found to infect pear and citrus in South Africa [12,13]. A recent study showed that the virus was related to the impietratura symptoms of citrus by using high-throughput sequencing (HTS)for identification of the impietratura infectious agent along with a comparative analysis among symptomatic and asymptomatic orange fruits in Greece [14].

Pear (*Pyrus* spp.) is widely grown in China. During field investigations, viral-disease-like symptoms are frequently observed on pear trees grown in China. In a recent study, we molecularly characterized a pathogenic emaravirus named pear chlorotic leaf spot associated virus (PCLSaV), which is associated with pear chlorotic leaf spot (PCLS) disease [15]. Meanwhile, leaf mosaic, chlorotic rings, and vein yellowing diseases also occur on some pear trees. To investigate the virome in the viral-disease-affected pear trees, leaves showing mosaic or chlorotic symptoms were collected from four pear trees, and subjected to RNA sequencing (RNA-Seq) and bioinformatic analyses. In addition to PCLSaV, two other -ssRNA viruses ARWV-2 and CiVA were identified. This is the first finding of these two viruses infecting pear grown in China. Here, we report the complete genome sequences of ARWV-2 and CiVA infecting pear by using high throughput sequencing combined with conventional Sanger sequencing and their infection status in pear trees grown in China. In addition, for the first time the subcellular localization of the predicated movement proteins encoded by the two viruses was determined.

## 2. Materials and Methods

### 2.1. RNA Sequencing

**Figure 1:**
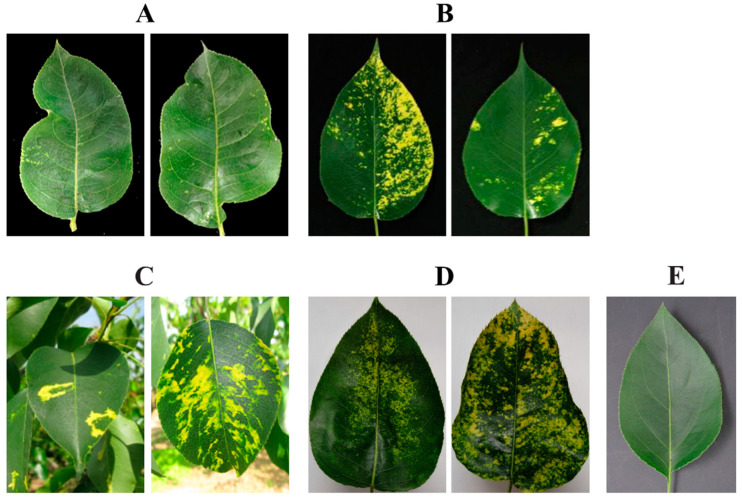
Disease symptoms on pear leaves used for high-throughput sequencing. (**A**) Cuiyu (ID: S17E2), (**B**) Chili (ID: LYC2), (**C**) Xiangshui (ID: LYXS), (**D**) Cuiguan (ID: FJCG), and (**E**) Huanghua (ID: FJHH).

A leaf sample (ID: S17E2) from a *P. pyrifolia* cv. Cuiguan tree, from which ARWV-2 was identified, as reported previously [5], was included in this analysis. Samples LYC2 and LYXS were individually collected from a *P. bretschneideri* cv. Chili tree and a *P. bretschneideri* cv. Xiangshui tree grown in the Laiyang area, Shandong Province, China. The leaf samples FJCG and FJHH were individually collected from a *P. pyrifolia* cv. Cuiguan and a *P. pyrifolia* cv. Huanghua grown in Fujian Province, China. The three trees S17E2, LYC2, and LYXS exhibited brilliant yellow mosaic symptoms and yellow rings on some leaves of LYXS, the FJCG plant exhibited symptoms of chlorotic mottle and vein clearing, and the FJHH plant exhibited no obvious symptoms (Figure 1). These five samples were used for RNA-Seq analyses as described previously [15]. Briefly, ribosomal RNA (rRNA) in total RNA extracts was removed using an Epicentre Ribo-ZeroTM rRNA removal kit (Epicentre, Madison, WI, USA). The prepared rRNA-depleted RNA sample was used to construct a cDNA library with a TruSeq RNA Sample Prep Kit v2 (Illumina, San Diego, CA, USA) and sequenced on an Illumina HiSeq XTen sequencing machine (Illumina, San Diego, CA, USA) with a paired-end 150 bp setup (Biomarker Biology Technology Ltd. Company, Beijing, China). Analysis of the sequence data was performed with CLC Genomics Workbench V.10.1.1 (QIAGEN, Hilden, Germany).

The raw RNA reads from the Illumina platform were trimmed of adaptor sequences and filtered for low-quality reads using FASTP version 1.5.6 to remove adapter sequences and reads with more than 5% Ns or with 20% base quality values (Q20) less than 20. Then, the obtained RNA reads from each sample were de novo assembled into larger contigs using Velvet version 1.2.08 [16] with a k-mer of 15–17 and IDBA-UD version 1.1.1 [17] with k-mer values of 80, 90, and 110. Contigs were subsequently screened for sequence identities against the NCBI databases (http://www.ncbi.nlm.nih.gov, accessed on 16 October 2019) using BlastX and BlastN programs.

### 2.2. Amplification of the Viral Genomic RNAs

For the amplification of the CiVA genomic RNAs, cDNA templates were generated using random primer pd(N)6. Specific primers (Table S1) were designed based on the sequences of assembled contigs and their positions mapped to reported CiVA genomic RNAs (accession numbers. MG764565 and MG764566). For the amplification of the ARWV-2 genomic RNAs, the gaps in the RNA-Seq-derived contig sequences were filled by nested RT-PCR amplifications using primers designed based on contig sequences and reported genomic RNA sequences of ARWV-2 (MF062139–MF062143). For RNA1 amplification, total RNAs from ARWV-2-infected samples were reverse-transcribed using the random primer pd(N)6. For the amplification of M and S RNAs, cDNA templates were generated using primers targeting the 5′ termini of the viral RNAs (Table S1).

The terminal regions of the genomic RNAs of CiVA and ARWV-2 were determined by the RACE strategy using a commercial kit (GeneRacer, Invitrogen Carlsbad, CA USA) according to the manufacturer’s instructions. For 3′ RACE, poly (A) tails were added to the 3′ ends of the total RNAs by using the poly (A) polymerase kit (TaKaRa, Dalian, China), and cDNA was generated using the oligo (dT) primer provided in the 3′ RACE kit. For 5′ RACE, specific reverse primers were designed based on the nucleotide sequences conserved at the 5′ termini of the genomic RNAs of each virus (Table S1).

RT-PCR solutions and conditions were similar to those reported previously [15], except that annealing temperature and extension time varied depending on the primer sets used in each reaction and the size of the expected PCR products. PCR products were gel-purified and ligated into the pMD18-T vector (TaKaRa, Dalian, China). At least three positive clones of each PCR product were sequenced at Shanghai Sangon Biological Engineering & Technology and Service Co. Ltd., Shanghai, China. The obtained sequences were assembled into contiguous sequences by overlapping common regions of the amplicons.

### 2.3. Sequence Analyses

Prediction of ORFs was performed using the NCBI ORFFinder program (https://www.ncbi.nlm.nih.gov/orffinder, accessed on 12 July 2021) with minimal ORF length of 75 nt. Multiple sequence alignments and identity analyses were performed using the Muscle method in MEGA 7.0 [18]. Phylogenetic trees were constructed using the neighbor-joining method with 1000 bootstrap replicates. The corresponding sequences of an ARWV-1 isolate (accession numbers: MF062130, MF062131, and MF062132) and a CCGaV isolate (accession numbers. KX960112 and KX960111) were used as outgroups in the ARWV-2 and CiVA sequence-based trees. The numbers of mapped reads in virus genomic RNAs were measured by Samtools version 1.5 [15] with the default parameters. The read coverage was calculated using software Fastv version 1.0 [19] with the default parameters. The conserved protein domains were identified using the Pfam database [20] with the default parameters.

### 2.4. RT-PCR for Virus Detection

To understand the infection status of ARWV-2, leaf samples were collected from 173 pear trees grown in five provinces in China. Of these samples, 99 samples showed chlorotic leaf spots or yellow mosaic symptoms and 74 samples were asymptomatic. Total RNA extraction and reverse transcription (RT) were done as described above. For the efficient detection of ARWV-2, a nested RT-PCR (RT-nPCR) method was developed by using SaF1/SaR1 as an outer primer set and NP1-F/NP1-R as an inner primer set. The two sets of primers were designed in the sequence of ARWV-2 Sa segment (Table S1). The RT-nPCR solutions and conditions were similar to those reported previously [21]. For the detection of CiVA, a primer set R2-F/R2-R was designed from the sequence of CiVA ORF2b (Table S1). In these tests, a leaf sample of a virus-free seedling of *P. betuleafolia* was used as a negative control. PCR products were separated by electrophoresis on 2% agarose gels, stained with ethidium bromide, and visualized under UV light.

### 2.5. Subcellular Localization and Bimolecular Fluorescence Complementation Analysis

Two ORFs (without stop codons) coding for Ma and Mb of ARWV-2 isolate LYXS, and ORFs (without stop codons) coding for NP and MP of CiVA were amplified using gene-specific primers flanked with an *attB* recombination sequence (Table S2). The construction of vectors used for protein localization and bimolecular fluorescence complementation (BiFC) assays were done as described previously [22].

Recombinant plasmids were individually transformed into *Agrobacterium tumefaciens* strain GV3101 (Weidi Bio, Shanghai, China), and agro-infiltrated into leaves of *Nicotiana benthamiana* plants (5 weeks old). The CMV3a-mCherry and H2B-mCherry vectors were used as plasmodesma (PD) and nuclear markers, respectively. Fluorescence signals of fusion proteins transiently expressed in infiltrated leaves of *N. benthamiana* plants were viewed at 2 days post infiltration (dpi) using confocal laser scanning microscopy (CLSM; TCS-SP8, Leica Microsystems, Heidelberg, Germany) with an HC PL APO CS2 63×/1.20 WATER objective.

## 3. Results

### 3.1. RNA-Seq of ARWV-2 and CiVA

Totally, 87,218,776, 69,655,848, 76,050,182, 83,312,860, and 124,323,162 clean reads were obtained from samples S17E2, LYC2, LYXS, FJCG, and FJHH, respectively. BlastN and BlastX searches against the NCBI database using assembled contigs revealed plant viruses presenting in these samples. From samples S17E2 and LYC2, three contigs with lengths of 381–1499 bp and six contigs with lengths of 261–475 bp matched sequences of ARWV-2 genomic RNAs named L, Ma, and Sa. From sample LYXS, six contigs with lengths of 282–7367 bp matched sequences of genomic RNA segments L, Ma, Mb, Sa, and Sb of ARWV-2 isolates 982-11, R7, and R12. From sample FJCG, two contigs with lengths of 6703 bp and 2765 bp covering near 95% of CiVA genomic RNA1 and RNA2 were identified. From sample FJHH, five contigs with lengths of 406–4597 bp matched sequences of CiVA genomic RNA1 and RNA2. Meanwhile, two well-documented viruses, apple stem pitting virus (ASPV) and apple chlorotic leafspot virus (ACLSV), were identified in the sample S17E2, and apple stem grooving virus (ASGV) and ASPV were identified in the samples LYC2 and LYXS. The specimens FJCG and FJHH were infected with ASPV, ASGV, and ACLSV. Here, we considered only the two -ssRNA viruses ARWV-2 and CiVA for further analyzes. The identified contigs of ARWV-2 and CiVA are presented in Table 1.

Complete and near-complete sequences of ARWV-2 genomic RNAs from the three samples S17E2, LYC2, and LYXS, and complete genomic sequences (GenBank accession numbers: MZ819702–MZ819703) of CiVA from the sample FJCG were reconstructed by Sanger sequencing of RT-PCR products. The reconstructed genomic sequences showed over 99% identities with corresponding contig sequences generated from RNA assembly, indicating that the assembled contig sequences from RNA-Seq data were reliable.

### 3.2. Genomic Characteristics and Sequence Diversity of ARWV-2 Isolates Infecting Pear

The genome structures of ARWV-2 determined from samples S17E2 and LYC2, here named isolates S17E2 and LYC2, were identical to those of reported isolates BR-Gala, 355-1, and R7, and consisted of three RNA segments L, Ma, and Sa. The genome structure of ARWV-2 from sample LYXS, here named isolate LYXS, was identical to that of reported isolate R12, and consisted of five RNA segments L, Ma, Mb, Sa, and Sb. Complete sequences of L, Ma, and Sa segments of isolate S17E2 were 7370 nt, 1594 nt, and 1486 nt (GenBank accession numbers: MN163133–MN163135). For isolate LYC2, a near full-length L sequence (6736 nt, 314–7049 nt), a full-length Ma sequence (1607 nt, excluding 19 nt at 5′terminal), and a near full-length Sa sequence (1387 nt, excluding 127 nt at 3′terminal) (GenBank accession numbers: MZ819704–MZ819706) were determined. For isolate LYXS, a near full-length L sequence (7366 nt, excluding 127 nt at 3′terminal) and Ma sequence (1547 nt, excluding 61 nt at 3′terminal), a full-length Mb sequence (1602 nt), a full-length Sa sequence (1510 nt), and a near full-length Sb sequence (1243 nt, excluding 75 nt at 3′terminal) (GenBank accession numbers: MZ819707–MZ819711) were determined. The obtained sequences, together with the corresponding sequences of reported ARWV-2 isolates, were comparatively analyzed (Table 2). The genome-wide nt sequence identities among the ARWV-2 isolates were about 93.6–99.5%, 97.7–99.3%, 97.7–98.1%, 96.4–99.5%, and 97.1% for their ORFs in the RNA segments L, Ma, Mb, Sa, and Sb, respectively. Although the size of each segment was variable among isolates, except for the size variation occurring in Sa-ORF, other ORF sizes were the same among ARWV-2 isolates. Each ORF of isolate LYXS had the same size as the corresponding ORF of isolate R12. The size difference of each segment among these isolates was a result of length variability of the 5′untranslated region (UTR). The L segment of isolates LYXS, 355-1, R7, 982-11, and BR-Gala had a 30-nt insert as compared with isolate R12 (Figure S1). There were also different inserts or deletions in the 5′UTRs of other RNA segments of these isolates (Figure S1). Although both isolates LYXS and R12 had five segments, the inserts or deletions in the 5′UTRs of each RNA segment of the two isolates were not conserved. Most of the inserts occurred at the “A” enriched regions (Figure S1).

**Figure 2:**
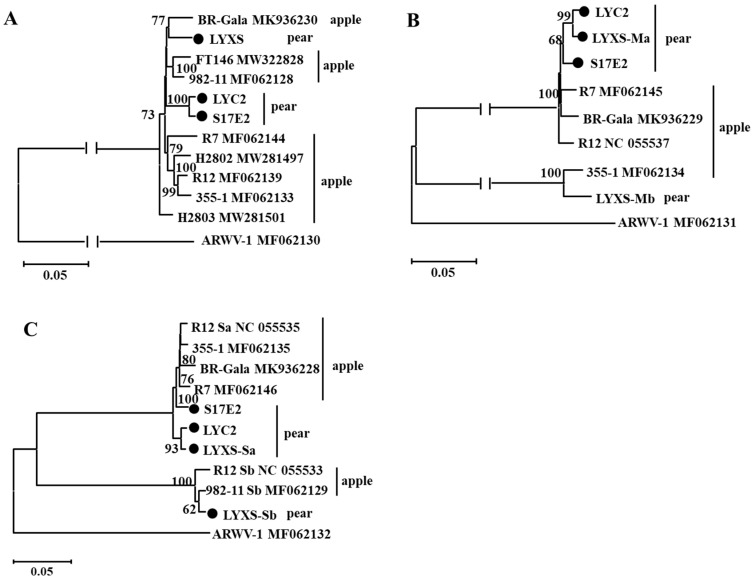
Unrooted NJ phylogenetic trees generated from the nucleotide sequences of L (**A**), M (**B**) and S (**C**) RNA segments of ARWV-2. In the trees based on M (**B**) and S (**C**) segment sequences, Ma and Mb, and Sa and Sb sequences from pear LYXS and the two reported isolates were included in the assay. The corresponding sequence of a reported ARWV-1 isolate was used as an outgroup in each tree. The referred sequences are marked by their isolate names followed by GenBank accession numbers. Three ARWV-2 isolates determined by high throughput sequencing are highlighted by black dots.

Neighbor-joining (NJ) trees constructed using the nucleotide sequences of the viral RNA segment L showed that the three ARWV-2 isolates from Chinese pear samples clustered into a large clade with the previously reported seven ARWV-2 isolates, and one isolate H2803 (host: apple) from the USA formed a separated clade. In the trees of segments M and S, the three Chinese ARWV-2 isolates clustered into two clades, with Mb and Sb segments of LYXS separated from M and S segments of S17E2 and LYC2, respectively (Figure 2).

### 3.3. Genomic Characteristics of CiVA

By RT-PCR amplifications and 3′ RACE reactions, the full-length sequences of the viral RNA1 and RNA2 of CiVA from pear sample FJCG (here named as isolate CiVA-P) were determined. Moreover, an expected amplicon with a size 1973 bp was obtained by using a primer pair N155F/M240R designed based on the sequences of the covering partial sequences of the two ORFs in the viral RNA2. The result confirmed that the ORF2a and ORF2b were encoded in the same RNA strand. The nt sequences of CiVA-P RNA1 and RNA2 obtained by Sanger sequencing were 99.9% and 99.6% identical to the corresponding contig sequences generated by *de novo* assembly, respectively. The overall nt identities between the isolate CiVA-P from a Chinese pear sample and each of the nine isolates available in GenBank were about 95% for their RNA1 and 93–94% for their RNA2. Among these CiVA isolates, the intergenic region (IGR) between ORF2a and ORF2b ranged from 301 nt to 313 nt, and CiVA-P determined here showed relatively low nt identities with other isolates reported from other countries, ranging from 83.9% to 88.0% (Table 3). The IGR had a high AU content of 76% and could form a secondary structure with a stop signal CUCUGCU conserved in phleboviruses [23].

The CiVA-FJCG RNA1 was 6690 nt in length with one ORF (ORF1, 6640–86 nt) in its complementary strand. The ORF1 encoded a putative RdRp consisting of 2184 aa with a molecular mass of 251 kDa, which contained six motifs conserved in the RdRp of viruses in the *Bunyaviriles* [24]. ORF1 of CiVA-P isolate shared 94.6–95.3% nt and 95.9–96.9% aa identities with the corresponding sequences of other CiVA isolates (Table 3). The RNA2 of CiVA-P was 2739 nt in length and contained two ORFs in opposite directions. ORF2a (57–1224 nt) encodes a MP of 395 aa with a molecular mass of 44.4 kDa. ORF2b (2667–1555 nt) encodes a NP of 370 aa with a molecular mass of 41.8 kDa. Its ORF2a shared 94.3–95.6% nt and 93.5–98.2% aa identities with the corresponding sequences of other CiVA isolates (Table 3). Its OR2b showed 94.0–95.0% nt and 93.0–95.7% aa identities with the corresponding sequences of other CiVA isolates.

**Figure 3:**
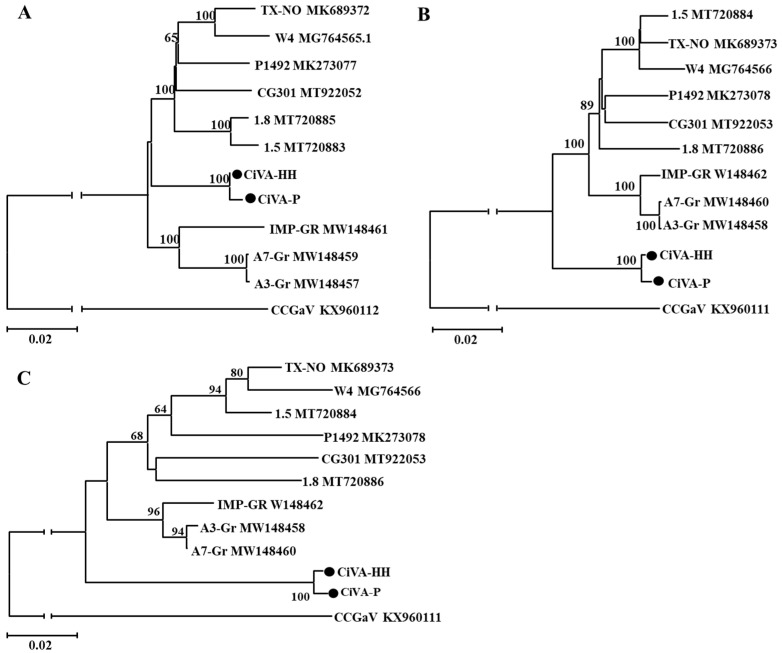
Unrooted NJ phylogenetic trees generated from the nucleotide sequences of RNA1 (**A**), RNA2 (**B**) segments of CiVA. The intergenic region (IGR) (**C**) sequences of CiVA RNA2 were also used for phylogenetic analysis. The corresponding sequence of a reported CCGaVisolate was used as an outgroup in each tree. The referred sequences are marked by their isolate names followed by GenBank accession numbers. Two Chinese CiVA isolates determined by high throughput sequencing are highlighted by black dots.

In the NJ trees constructed using the nucleotide sequences of the viral RNA1, RNA2, and IGR, two Chinses isolates CiVA-P and CiVA-HH were always separated from other-CiVA isolates reported from other countries, and formed a distinct subclade, indicating genetic diverse of CiVA isolates CiVA-P and CiVA-HH, identified from pear grown in China, from other reported CiVA isolates (Figure 3).

### 3.4. RNA Profiles of Viruses ARWV-2 and CiVA

In total, 251 reads, 77 reads, and 12,911 reads matching ARWV-2 genomic RNAs were derived from samples S17E2, LYC2, and LYXS and 90,617 CiVA RNA reads were derived from sample FJCG. The high RNA read productivities of ARWV-2 from LYXS and CiVA from FJCG made it possible to evaluate the distribution of RNA reads on each segment of the two viral isolates. It was found that ARWV-2 RNA reads deriving from sample LYXS covered the full lengths of the five RNA segments (L, Ma, Mb, Sa, and Sb) of the viral genome (Figure S2). On each RNA segment, the RNA reads distributed unevenly, with several peaks at different positions. In particular, a prevalent read peak appeared in the 5′UTR of the Ma segment. The RNA read depths also differed among the RNA segments, with relatively high depths occurring in Sb and Mb segments (Figure S2).

CiVA RNA reads derived from sample FJCG also covered the full lengths of the viral RNAs 1 and 2. The overall read abundance on the viral genomic RNA1 was much lower than that of the viral genomic RNA2 (Figure S2). RNA reads of CiVA separated almost evenly in the viral genomic RNA1 except for several hotspots, whilst, RNA reads on the viral genomic RNA2 had two prevalent read peaks separated by an IGR.

### 3.5. Subcellular Localization of ARWV-2 and CiVA Proteins in Planta

**Figure 4:**
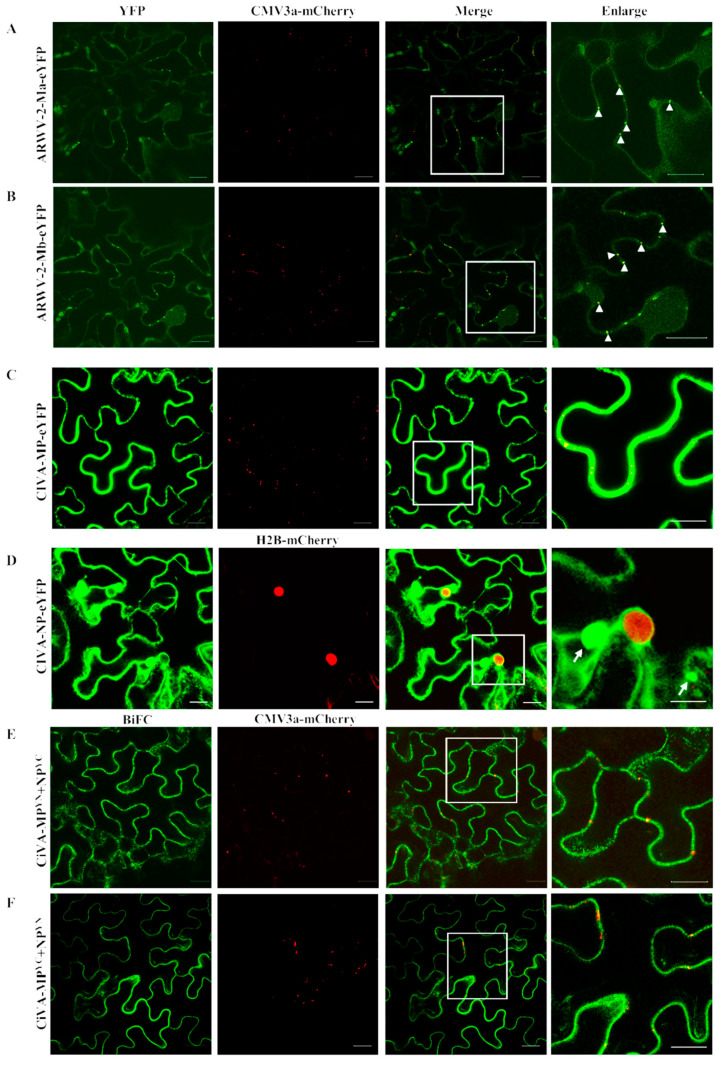
Subcellular localization (**A**–**D**) and bimolecular fluorescence (BiFC) (**E**,**F**) assays of two movement proteins Ma and Mb of ARWV-2 and MP and NP of CiVA in epidermal cells of wild-type *Nicotiana benthamiana* leaves. The fusion proteins CMV3a-mCherry and H2B-mCherry were used as plasmodesmata (PD) and nuclear markers, respectively. Colocalization dots of Ma and Mb with CMV3a-mCherry at PD are indicated by arrow heads (**A**,**B**). The aggregated bodies formed by protein NP of CiVA are denoted by arrows. The images were acquired 2 days after agroinfiltration under a confocal microscope at 63x/1.20 WATER objective. Scale bar = 20 μm.

Some ARWV-2 isolates have two additional RNAs coding proteins Sb and Mb, which show over 60% aa sequence identity with Sa and Ma. It was postulated that both Ma and Mb might function in the virus movement [1]. Pfam analysis showed that both protein Ma (interval: 123–236, E-value: 3.8 × 10^−12^) and Mb (interval: 130–242, E-value: 8.11 × 10^−7^) of ARWV-2 isolate LYXS contained the plant viral MP family (pfam01107) domains (Figure S3). To have a primary look at the subcellular localizations of the two proteins, the Ma and Mb were transiently expressed in epidermal cells of *N. benthamiana* leaves by using an agrobacterium infiltration method. It was found that proteins Ma and Mb showed a similar localization profile with fluorescence signals in periphery and punctate spots along cell membrane, which were co-localized with the plasmodesma (PD) marker CMV-3a-mCherry (Figure 4A,B), indicating that the viral Ma and Mb had the typical subcellular localization features of the movement proteins of plant viruses.

Although a core 30K viral MP domain containing a LxD/N_50-70_G motif was recognized in the MP of CiVA [8], whether the MP has the location features of viral movement proteins is unknown. This study showed that the fusion protein MP-eYFP of CiVA distributed smoothly along cell membrane and periphery, while the PD marker CMV-3a-mCherry distributed as punctate spots (Figure 4C). Considering that the cell to cell movement of some plant viruses needs MP and structure protein(s), we further localized the viral NP and tested the interaction between the viral NP and MP by BiFC assays. Results showed that NP-eYFP located in periphery and formed aggregates (Figure 4D), which looked like viral replication complexes (VRC) [25]. BiFC results showed that proteins NP and MP interacted with each other in both orientations. The interaction signal was observed along the cell periphery (Figure 4E,F), which was similar to the distribution of the viral MP, but different from PD marker CMV-3a-mCherry distribution.

### 3.6. RT-PCR Assays for ARWV-2 and CiVA in Pear Trees

RT-nPCR assays showed that ARWV-2 presented in 34 symptomatic and 24 asymptomatic samples, accounting for 34.3% and 32.4% of the 173 tested samples, respectively. The virus was detected in *P. communis*, *P. bretschneideri*, *P. pyrifolia,* and some hybrids (Table 4). In particular, 6 of 10 tested *P. communis* samples were positive to ARWV-2, with an incidence of 60%. Leaf samples from 57 pear trees (including the sample FJCG and FJHH used for the RNA-Seq analysis) grown in Hubei, Sichuan, and Fujian provinces in China were subjected to RT-PCR assay for CiVA using primer set R2-F/R2-R (Table S1). The virus was detected in four samples, accounting for 7.0% of the tested samples (Table 4). Of the CiVA-positive samples, one showed leaf mottle and two showed chlorotic leaf spot symptoms.

**Figure 5:**
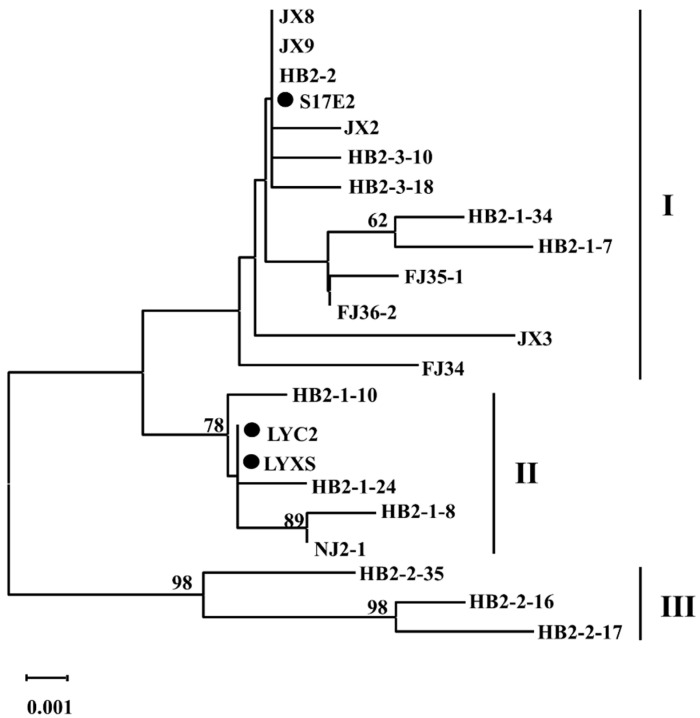
Unrooted NJ phylogenetic trees generated from the nucleotide sequences of partial S segment of ARWV-2. The corresponding sequences of three isolates S17E2, LYC2, and LYXS were included in the assay.

The 486-bp amplicons of ARWV-2 from 19 samples were cloned and sequenced. It was found that these amplified sequences shared about 99% nt and aa similarities and clones from each sample had over 99% nt similarity. The result indicated low sequence variation of the NP gene among ARWV-2 isolates. These sequences and the corresponding sequences of the three HTS detected isolates LYC2, LYXS, and S17E2 clustered into three distinct phylogenetic clades, with clade I represented with S17E2, clade II represented with LYC2 and LYXS, and the clade III consisting three isolates from Hubei Province (Figure 5).

## 4. Discussion

In this study, HTS was used to identify viruses in four diseased pear samples and revealed the presence of two recently described -ssRNA viruses, ARWV-2 and CiVA, and also three common pear-infecting viruses in the samples. This is the first report of the complete genome sequences of the two viruses from pear trees and the first detection of CiVA in China. The presented results extend our knowledge of the molecular characteristics and geographical distribution of the two viruses. The coverages of RNA-Seq reads on ARWV-2 genomic RNA segments varied among samples. The near full-length sequences of five RNA segments of ARWV-2 were assembled from the LYXS sample, which showed very strong mosaic disease symptom, while from sample LYC2, the ARWV-2 RNA read coverage was very low, which resulted in short assembled contigs (less than 500 nt). From sample S17E2, near full-length sequences of Sa and Ma segments were assembled, but only one short contig of 148 bp of the viral L segment was identified. CiVA reads had high cover depth and near full-length sequences of CiVA genomic RNA1 and RNA2 were assembled from FJCG library. HTS technologies have been proved to be a powerful tool for the identification of known and novel viruses [26,27]. Since the quality of these libraries were reliable with QC20 and QC30 values of 94%–99%, the differences in viral RNA read coverages among samples could be caused by the virus titer or the quality of RNA-Seq libraries [28,29]. Interestingly, the RNA read profiles of Mb and Sb segments of ARWV-2 isolate LYXS were significantly higher than those of their homolog Ma and Sa segments. The biological functions of the redundancy proteins encoded in Ma and Mb, and Sa and Sb segments are still unknown [1]. The high distribution profiles of RNA reads on the viral Mb and Sb segments might be related to the genome expression strategy and involved in specific functions. Similarly, RNA segments encoding for redundancy proteins have been reported in several emaraviruses [15,22,30]. The RNA read peaks on the genomic RNA2 of CiVA are separated by an IGR, which is similar to the characteristics for some other viruses previously reported [31].

The overall genomic structures of ARWV-2 and CiVA determined from pear plants grown in China were the same as those of the corresponding viruses infecting apple [1] and citrus [11], as reported previously. The three pear isolates of ARWV-2 identified in this study showed over 97% genomic identity with each other and with reported apple isolates. One isolate LYXS had five RNA segments, which were identical to those of an apple isolate R12 [1]. Our results revealed that the size of each RNA segment was variable among ARWV-2 isolates as a result of their 5′-UTR variability. Except for sample LYXS, we did not detect the Mb and Sb segments of ARWV-2 from other pear samples, indicating a low occurrence frequency of the ARWV-2 Mb and Sb segments in pear grown in China. The complete genomic sequences of three CiVA isolates from citrus [8,12], two CiVA isolates from apple [14], and a near-completed genomic sequence of two CiVA isolates from pear have been reported recently [11,13]. Here, we reported the complete genomic sequence of a CiVA-P isolate from a pear tree grown in China. The overall nt identities between the CiVA-P and each of CiVA isolates available in GenBank were about 95% for their RNA1 and 93–94% for their RNA2. Among these isolates, IGR between ORF2a and ORF2b had a size of 309 nt or 310 nt, and showed nt sequence diversity ranging from 11.4% to 13.8%. The sequence variation might be involved in the adaption of the virus in different host plants [32,33].

ARWV-2 was originally described in apple trees with rubbery wood disease [1]. In recent studies, the virus was detected in apple trees showing decline disease in the United States, asymptomatic trees from a global apple collection [7], and apple trees grown in China [4]. The latent infection of an apple rubbery-wood-disease-associated agent in pear has been recognized for some time [34]. In the present study, RT-nPCR assays showed that ARWV-2 commonly occurred in the investigated *P. communis*, *P. bretschneideri*, *P. pyrifolia,* and pear hybrids grown in several Chinese provinces. Although ARWV-2 was first detected by HTS in three pear trees showing leaf mosaic symptom, the virus also presented in some asymptomatic trees as detected by using RT-nPCR. Our previous bioassays demonstrated the infectious nature of ARWV-2 in pear plants, but did not find the associated symptoms on pear A20 [5]. The leaf mosaic symptom of the three RNA-Seq-analyzed samples was different from the chlorotic leafspot disease caused by PCLSaV [15], but like the mosaic symptom in apple trees infected by apple necrotic mosaic virus (ApNMV) [35]. However, we did not identify either PCLSaV or ApNMV by HTS in the samples. Further studies are needed to evaluate viruses potentially associated with the observed leaf mosaic disease. CiVA was originally characterized from an asymptomatic orange leaf sample [8]. Recently, the virus was found to be associated with impietratura disease of citrus in Greece [14]. In this study, we found that CiVA occurred only in four *P. pyrifolia* trees grown in Fujian and Sichuan provinces, indicating a lower incidence. Although CiVA was identified in pear trees showing viral-disease-like symptoms (one leaf mottle and two chlorotic leafspot), we couldn’t conclude the association of the virus with pear disease since the most tested samples showing similar symptoms were negative to the virus. In addition, we did not find the co-infection of the two viruses.

Considering the close taxon position of apple and pear belonging to the family Rosaceae, and the common infection of previously characterized viruses and viroids, including ASGV, ASPV, ACLSV, and apple scar skin viroid (ASSVd) [36], together with the two -ssRNA viruses characterized here, it is reasonable to speculate that similar virus origins might have occurred in apple and pear. Vegetative propagation has been proposed to play prevalent roles for the transmission of viruses and other graft-transmissible agents of perennial fruit trees. The wide infection of ARWV-2 as well as the three common viruses ASGV, ASPV, and ACLSV and one common viroid ASSVd indicate a relatively long occurring history of these viruses. The low occurrence frequency of CiVA might be a result of its recent introduction.

The 30K family MPs can form MP–RNA complexes to facilitate cell to cell movement of viral genome RNA via the PD [1]. Here, for the first time, we showed that two proteins Ma and Mb of ARWV-2 isolate LYXS displayed PD location, further supporting that the proteins might co-function on the virus movement [37,38]. However, the predicated MP of CiVA showed a cytoplasm location, which was different from the typical PD location of plant virus movement proteins, but similar to the locations of the movement protein NSm of two orthotospoviruses, impatiens necrotic spot (INSV), and iris yellow spot (IYSV) [39,40]. Likely, the movement functions of some plant virus proteins might not always be correlated with PD location [41,42]. Similar to nucleocapsid proteins of several orthotospoviruses, including capsicum chlorosis virus (CaCV), tomato spotted wilt virus (TSWV), INSV, and IYSV [25,39,40,42], the CiVA protein NP formed VRC-like aggregates in the cytoplasm of agroinfiltrated cells. The NP–MP complexes of orthotospoviruses are involved in ribonucleoprotein (RNP) movement to adjacent cells [43]. Thus, the interaction of CiVA proteins NP and MP might be necessary for the virus movement.

In conclusion, ARWV-2 and CiVA were identified in pear trees grown in China using HTS. The genome of ARWV-2 pear isolates is divergent, with three RNA segments or five RNA segments. ARWV-2 occurs widely, but CiVA has low incidence in pear trees grown in China. ARWV-2 isolate LYXS codes two movement proteins Ma and Mb, which display PD localization. The MP of CiVA is located in cell periphery and can interact with the viral NP.

## Figures and Tables

**Table 1:** Blast analysis of contigs derived by RNA-seq from five pear samples.

Sample	RNA	Contig (bp)	Matched Sequence	nt		aa	
				Site	%	Site	%
S17E2	L	381 ^a^	NC_055534.1	2224–2373, 2457–2604	97.0	721–771, 791–847	100
	Ma	1430	MF062145.1	14–1431	97.1	1–370	99.7
	Sa	1499	MF062146.1	5–1237	96.9	1–370	98.4
LYC2	L	261	MW322828.1	7056–7315	97.3	2331–2376	100
		321	MF062133.1	5732–6012	99.1	1884–1989	99.1
		326	MW322828.1	5366–5691	99.4	1768–1865	99.1
	Sa	326	MF062146.1	444–769	98.5	126–233	98.2
		288	MK936228.1	156–443	99.3	34–104	100
	Ma	475	NC_055536.1	817–1291	96.4	253–370	94.9
LYXS	L	7367	MF062128.1	1–7366	98.3	1–2376	98.2
		282	MF062128.1	3466–3746	100	1135–1228	100
	Sb	1316	NC_055533.1	16–1316	98.0	1–286	98.6
	Sa	1511	MF062146.1	1–1488	96.8	1–288	98.6
	Mb	1611	NC_055537.1	1–1608	97.9	1–393	98.5
	Ma	1461	MF062145.1	1–1437	96.0	1–370	98.4
FJCG	RNA1	6703	MT922052.1	14–6679	95.3	1–2184	96.8
	RNA2	2765	MT720884.1	4–2736	94.4	1–395 (MP)	97.9
						1–370 (NP)	94.0
FJHH	RNA1	661	MT922052.1	1–563	94.1	202–2184	94.9
		1722	MT922052.1	456–2186	95.2	1486–2058	96.3
		4597	MK273077.1	2088–6684	95.6	45–4595	97.6
	RNA2	2562	MT720884.1	9–2530	94.1	1–395 (MP)	97.6
						47–370 (NP)	94.1
		406	MK273078.1	1888–2194	96.0	1–78	95.0

^a^ In the contig, 84 nucleotides between 2373 and 2457 nt were absent.

**Table 2:** RNA sequence comparison of ARWV-2 isolates with that of an isolate LYXS determined in this study.

Host	Isolate ^a^	ORF1 (L)		ORF2a (Ma)		ORF2b (Mb)		ORF3a (Sa)		ORF3b (Sb)	
		nt	nt%	nt	nt%	nt	nt%	nt	nt%	nt	nt%
Pear	**LYXS**	7367	/	1134		1182	/	867	/	855	/
	**S17E2**	7370	97.6	1134	98	/	/	867	96.4	/	/
	**LYC2**	6736	99.5	1134	99.3	/	/	810 (partial)	99.5	/	/
Apple	R12	7340	97.4	1134	97.8	1182	98.1	867	98.6	861	97.1
	BR-Gala	7349	93.6	1134	97.7	/	/	867	98.7	/	/
	355-1	7381	97.4	/	/	1182	97.7	867	98.3	/	/
	R7	7369	97.3	1134	97.4	/	/	867	98.7	/	/

^a^ Isolates determined in this study are in bold.

**Table 3:** RNA sequence comparison of CiVA from two Chinese pear samples with that of reported CiVA isolates.

Host	Isolate ^a^	ORF1			ORF2a			IGR		ORF2b		
		nt	Nt %	aa %	nt	nt %	aa %	nt	nt %	nt	nt %	aa %
Pear	**FJCG**	6555	-	-	1188	-	-	310		1113	-	-
	HH	/	/	/	1118	95.6	98.2	313	98.7	/	/	/
	P1492	6555	95.3	96.8	/	94.3	93.5	310	85.3	1113	95.0	95.4
Citrus	CG301	6555	95.1	96.7	1188	95.5	97.0	309	84.4	1113	94.4	94.3
	TXNO	6555	94.9	96.5	1188	95.0	95.7	309	88.0	1113	94.1	93.5
	W4	6555	94.7	95.9	1188	94.6	95.4	310	86.6	1113	94.0	93.8
	1.8	6555	95.3	96.7	1188	94.8	95.6	301	84.1	1119	93.5	93.0
	1.5	6555	95.0	96.3	1188	94.9	94.9	309	87.4	1113	94.7	94.6
	IMP-GR	6555	94.6	96.3	1188	95.2	96.7	303	83.9	1113	94.2	95.4
	A7-Gr	6555	95.0	96.9	1188	95.2	96.7	303	87.3	1113	94.3	95.7
	A3-Gr	6555	94.9	96.8	1188	95.0	96.5	304	87.3	1113	94.3	95.4

^a^ The isolate determined in this study is in bold.

**Table 4:** Incidence of ARWV-2 and CiVA in the pear leaf samples of five provinces of China as detected by RT-PCR.

Virus	Species	No. of Samples	Infected/Tested	
			Asymptomatic	Symptomatic
ARWV-2	*P. communis*	10	5/9	1/1
	*P. bretschneideri*	21	8/19	1/2
	*P. pyrifolia*	116	11/45	25/71
	hybrids	22	0/1	7/21
	unknown	4	0	0/4
	total	173	24/74	34/99
CiVA	*P. pyrifolia*	25	0	3/25
	hybrids	0	0	0
	unknown	22	0	0
	total	57	0	0

## Data Availability

The data presented in this study are available in article and supplementary materials.

## Supplementary Materials

The following are available online at https://www.mdpi.com/article/10.3390/v14030576/s1, Figure S1. Multiple alignment of nucleotide sequences at the 5′un-translation region (5′UTR) of RNA segments of ARWV-2 amplified from pear samples and the corresponding sequences of reported isolates. Each sequence was labeled with a sample ID. Figure S2. Profile distribution of RNA reads from LYXS and FJCG along genomic RNAs of apple rubbery wood virus (ARWV-2) and citrus virus A (CiVA), respectively. The open reading frames were marked with green bold boxes under each RNA segment of ARWV-2 and CiVA. Figure S3. Pfam analysis the conserved domains of the proteins Ma (**a**) and Mb (**b**) encoded by ARWV-2. Table S1: Primers used for the genomic sequence amplification and RT-PCR detection of ARWV-2 and CiVA. Table S2: Primers designed for amplification the apple rubbery woody ARWV-2 ORFs of Ma and Mb and CiVA ORFs of NP and MP.
