# Supplementary material for: Molecular Characteristics and Incidence of Apple Rubbery Wood Virus 2 and Citrus Virus A Infecting Pear Trees in China
Source: Viruses. 2022 Mar 11;14(3):576. doi: 10.3390/v14030576 (PMC8952854; doi:10.3390/v14030576)
Supplement: Supplementary file 1 [file viruses-14-00576-s001.zip › Supplementary figures.pdf]

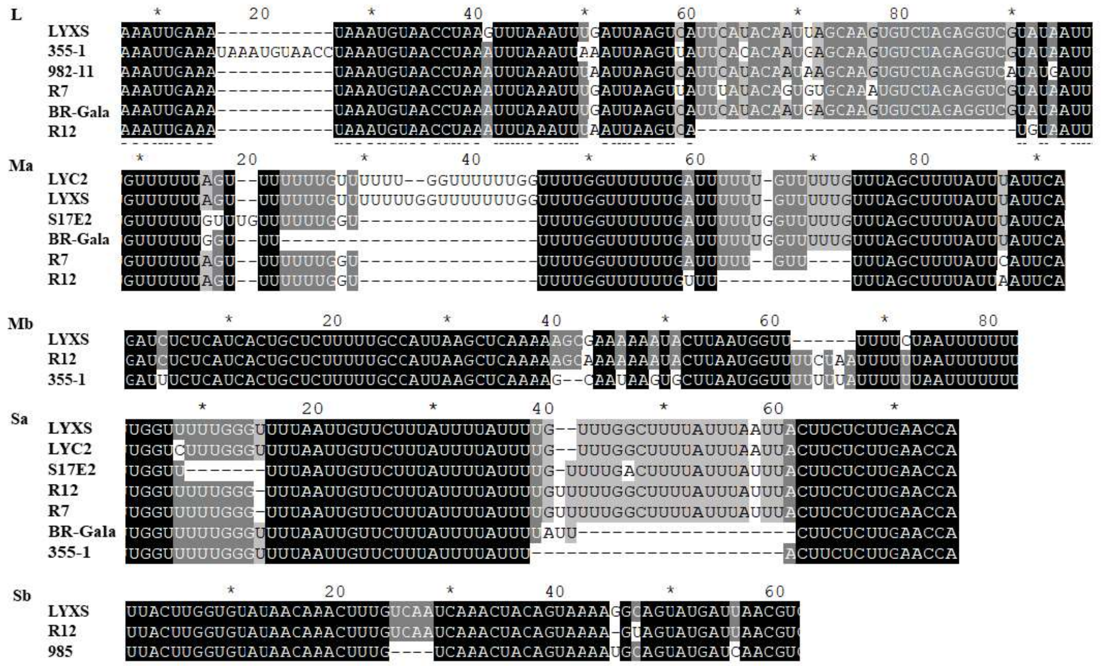

Figure S1. Multiple alignment of nucleotide sequences at the 5′ un-translation region (5'UTR) of RNA segments of apple rubbery wood virus 2 (ARWV-2) amplified from pear samples and the corresponding sequences of reported isolates. Each sequence was labeled with a sample ID.

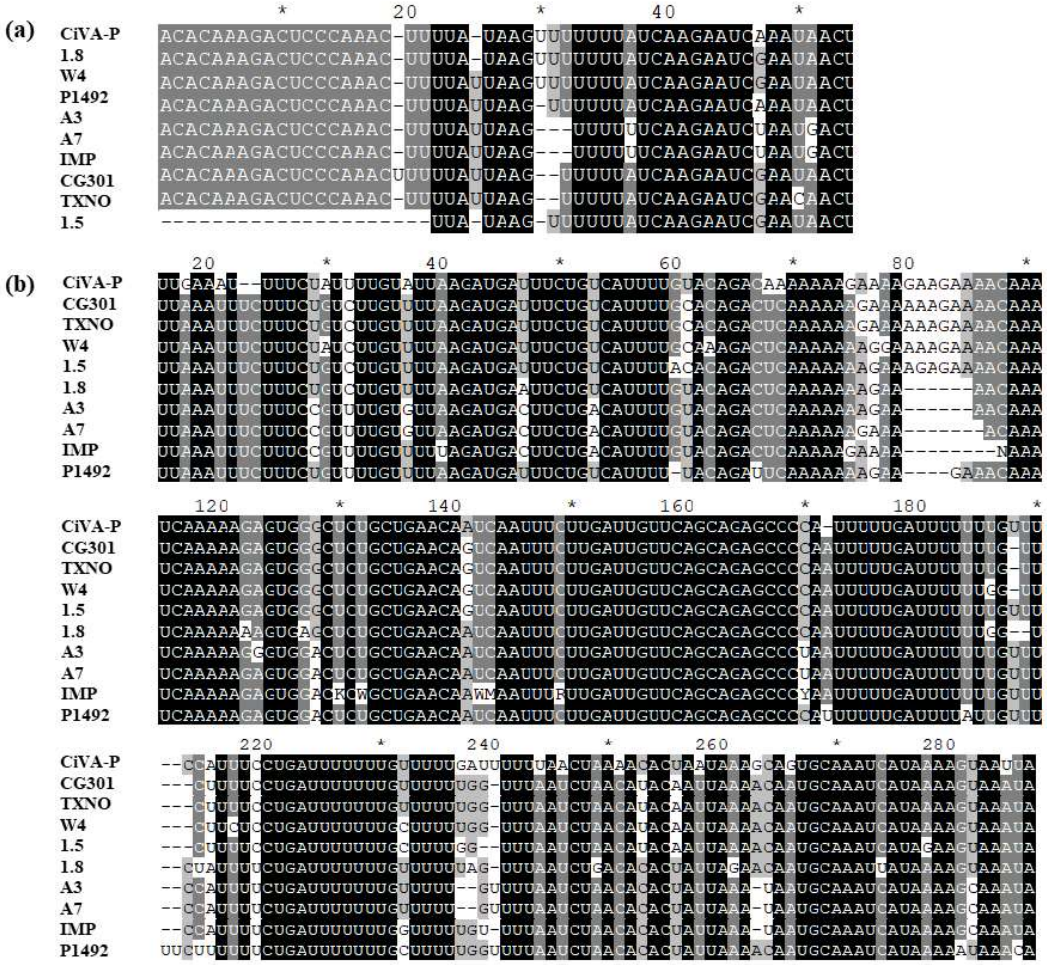

Figure S2. Multiple alignment of nucleotide sequences at the 5′ un-translation region (5'UTR) of RNA1 (a) and the IGR of RNA2 (b) of citrus virus A (CiVA) amplified from a pear sample FJCG and the corresponding sequences of reported isolates. Each sequence was labeled with a sample ID.

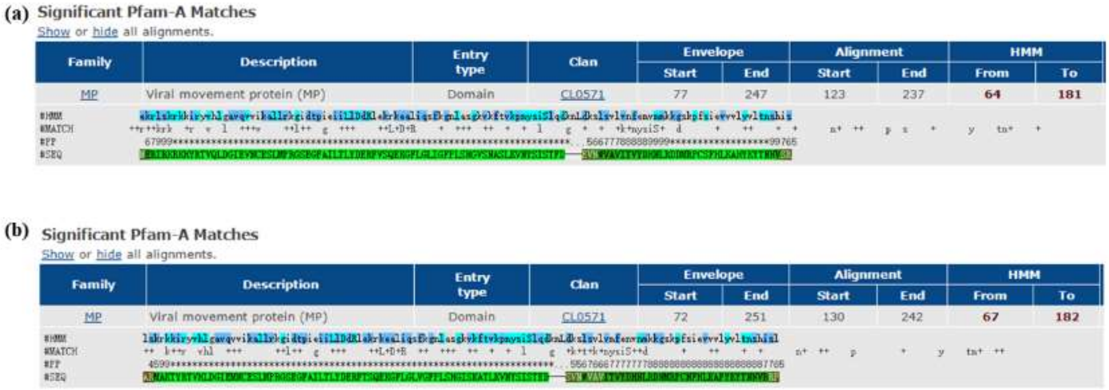

(a) Significant Pfam-A Matches

Show or hide all alignments.

Family	Description	Entry type	Clan	Envelope		Alignment		HMM	
				Start	End	Start	End	From	To
MP	Viral movement protein (MP)	Domain	CL0571	77	247	123	237	64	181

(b) Significant Pfam-A Matches

Show or hide all alignments.

Family	Description	Entry type	Clan	Envelope		Alignment		HMM	
				Start	End	Start	End	From	To
MP	Viral movement protein (MP)	Domain	CL0571	72	251	130	242	67	182

Figure S3. Pfam analysis the conserved domains of the proteins Ma (a) and Mb (b) encoding by apple rubbery wood virus 2 (ARWV-2).
