# Supplementary material for: Molecular Characteristics and Incidence of Apple Rubbery Wood Virus 2 and Citrus Virus A Infecting Pear Trees in China
Source: Viruses. 2022 Mar 11;14(3):576. doi: 10.3390/v14030576 (PMC8952854; doi:10.3390/v14030576)
Supplement: Supplementary file 1 [file viruses-14-00576-s001.zip › Supplementary tables.pdf]

Table S1. The primers used for the genomic sequence amplification and RT-PCR detection of apple rubbery woody virus 2 (ARWV-2) and citrus virus A (CiVA).

Virus	RNA	Primer[a]	Sequence (5'-3')	Position (nt)	Size (bp)
ARWV-2	L	LF1	AGGAGTATTTTCCTCTGATATGCC	7224-7247	807
		LR1	GCAATGAGGGAATTGACACAGG	6441-6462	
		LF1(2)	ACACCTGACTTTGTGAGTGAAG	7057-7036	578
		LR1(2)	CTAAACTCACCCTCTTTTCAGGAC	6480-6501	
		LF2	TTCCCTACTGAAGGCAGAACAG	6556-6535	1019
		LR2	CTCAAACTAGTAACTATCTC	5538-5557	
		LF2(2)	AGGCAGAACAGATCTTAAACGC	5545-5524	927
		LR2(2)	GCAGAATTATGACATCTATC	6619-6638	
		LF3	AGAGGTTTCAGGCAGATGTGT	5871-5751	1811
		LR3	GCCAAATTTGTATGAAAGACTG	4061-4082	
		LF3(2)	AGACCATTCGTAGCACATTCTGT	5679-5657	1393
		LR3(2)	TGGAAAAGACTTGATGAATGGTGC	4287-4310	
		LF4	TGAGCACAAATTTCCACACATCA	4434-4412	742
		LR4	ACAGCTGTTGCTACTATAGGTTC	3693-3715	
		LF4(2)	TCAGTCTGGATTCATGCAAGG	4337-4317	607
		LR4(2)	CAAAGATGGGTTTAAACATTC	3731-3751	
		LF5	GGCATTATCCCCAGTGCTGA	4063-4044	2086
		LR5	ATCCTAGTAAAATAAATTC	1258-1276	
		LF5(2)	GTTGCTTGCCAGTTGCCTTC	3349-3330	1848
		LR5(2)	TGTTCTTGCCAATCTTTTTAACTC	2102-2125	
		LF6	AAGTTAGAACTAGGGGATAATG	2362-2341	2223
		LR6	GCAAGTGTCTAGAGGTCGT	140-158	
		LF6(2)	TTGGCCTTGACCAATGGAAAC	2227-2207	1906
		LR6(2)	CTTGTCCCCATTCATCATTGC	322-342	
		L-RT	ACCCCTCCAAATTTCAAAAACACAT	1-25	450
		3′L-F	ACAACAACTTTTCTTCCATTGGC	428-450	
		5′L-F	ACCCCCTCCAACCTAAGCAT	7370-7351	311
		5′L-R	TGGGGACAAGAAGAGGACATTG	7040-7061	
	Ma	MaF1	GGAGACATTCCCTTTTCCGAAG	1473-1452	1057
		MaR1	TAGGCTTGTCAACATGTTTTTCCTC	417-441	
		MaF1（2）	GGAAAAGGGAGATTCTGGAACG	1418-1397	1418
		Ma-RT	GTTTTCCCAGTCACGACACCCCTCCACAACAGAG	1-17	
		3’M-F	GACTGCCACCCAATTCACTTC	679-659	659
		5’M-F	ACCCCCTCCACTTCAACTGGTA	1594-1573	756
		5’M-R	GGGCTTTGCAGAAGAGTCCAT	838-858	
	Mb	MbF1	AACTCCTTCTATCGAACAGC	1463-1444	1408
		MbR1	GATTTACGTCTGTTTGTATCC	56-76	

		MbF1 (2)	ACGCCTGAAGAATGACCCTG	1394-1375	1005
		MbR1 (2)	AATTTCCCCTTCATGGTTAAAGC	390-412	
		Mb-RT	GTTTTCCCAGTCACGACACCCCTCCACAA	1602-1591	-
	Sa	SaF1	TCTCACAACTGCTCTGACGC	1388-1369	1275
		SaR1	GCTTTGGGTCTTTGTGCCAG	114-133	
		SaF1(2)	CATCTCCTCACTGTTTCTG	1360-1342	1247
		Sa-RT	GTTTTCCCAGTCACGACACCCCTCCATAACCGAGA	1-17	-
		NP1-F	ACAAGGCAGTAGTTATTATCAGCA	861-885	486
		NP1-R	TTCTGCAACTAACTTCAAGGCTG	1346-1324	
		Sa-RT	-	1-17	329
		3'Sa-F	TCCTCTCGAATGTTCCCTCT	329-310	
	Sb	SbF1	CAACTGAATTCAAACGGTTTTTCTG	1295-1271	1261
		SbR1	TGTCAATCAAACTACAGTAAAAGGC	35-59	
		SbF1(2)	TCATCATCATGGCAAACGGTG	1243-1223	1166
		SbR1(2)	TTGTATCCAGAGCAAGGTTCGT	78-99	
		RT	GTTTTCCCAGTCACGACACCCCTCCAC	1-17	-
CiVA	RNA1	170F	GGAGCCAACTCTGTACGT	6578-6561	950
		1090R	ATGTCTTTACACCCATAGC	5629-5647	
		670F	GAGGCAATTGTCAAAGATGT	6049-6030	1192
		1890R	TCAATTCTTCGCAATCTG	4858-4875	
		1570F	GTTTGCTCTTTCAATGAACC	5034-5053	1514
		3320R	ATAATGTAATATTCCTTGCATC	3521-3542	
		2940F	CTTCATTCATGTAATGAGTCT	3731-3711	2051
		5130R	CATTTCCCTCTCCATTCTCC	1680-1699	
		4760F	TGCCAGGTAGGTCAGATG	2001-1984	1795
		6510R	CTATAAACAGATTGTTGTGC	207-226	
		5´L-R	GGTCTCAAAATTGGGTGCTAC	414-431	431
		RT	CCCGGATCCACACAHAGA	-	
		3´L-F	GATCCGGCTGACTTAGACTC	643-625	643
		3´L-R	TACCGTCGTTCCACTAGTGATTT	-	
	RNA2	N155F	CTAGAGTGTTTGATTATCAGG	2285-2265	1973
		M240R	TAAGTGGGAAAGATGAAGAG	313-332	
		3´S-F	TAGCAGGCTCACTCCTACCAC	2034-2054	706
		3´S-R	TACCGTCGTTCCACTAGTGATTT	-	
		5´S-R	TATGTATGGCCTGCCTTTC	488-470	488
		RT	CCCGGATCCACACAHAGA	-	
		R2-F	CCTCTGATTAATGATTAGTG	2218-2237	393
		R2-R	CTCTTCACCATTAGATGTTGC	2590-2610	

[a] Primers marked with (2) are used as nested or semi-nested primers of the corresponding outer primer set, primers marked with RT are used for reverse transcription, and primers marked with 3´ and 5´ were used for 3´- and 5´-race reactions, respectively. Underlined nucleotides mark sequences of no viral origin.

Table S2. Primers designed for amplification the apple rubbery woody virus 2 (ARWV-2) ORFs of Ma and Mb and citrus virus A (CiVA) ORFs of NP and MP.

Virus	Protein	Name	Sequence[a] (5´–3´)	Size (bp)
ARWV-2	Ma	Ma-F	AAAAAGCAGGCTCCATGGCTTTTACTATCTTTG	1131
		Ma-R	AGAAAGCTGGGTC TAACTTCAGTTTTCTAGG	
	Mb	Mb-F	AAAAAGCAGGCTCCATGACTTTTCTTGAAAAAC	1179
		Mb-R	AGAAAGCTGGGTC TATTTTCAATTGCTTGGG	
CiVA	NP	CP-F	AAAAAGCAGGCTCCATGGCACTCCACCAAAATC	1110
		CP-R	AGAAAGCTGGGTCAATATGAGTGGCATTG	
	MP	MP-F	AAAAAGCAGGCTCCATGTTTAAAAAGATGTTC	1185
		MP-R	AGAAAGCTGGGTCGACTCCAAACTCATTAAAC	

a: The artificially added nucleotides used for recombination reaction were underlined.
